# Synthesis and Properties of Cefixime Core–Shell Magnetic Nano-Molecularly Imprinted Materials

**DOI:** 10.3390/polym15224464

**Published:** 2023-11-20

**Authors:** Li Zhang, Hongbo Mo, Chuan Wang, Xiaofeng Li, Shuai Jiang, Weigang Fan, Yagang Zhang

**Affiliations:** 1 College of Chemistry and Chemical Engineering, Xinjiang Normal University, Urumqi 830054, China; 15099091024@163.com; 2 Chongqing Academy of Metrology and Quality Inspection, Chongqing 401123, China; 3 School of Materials and Energy, University of Electronic Science and Technology, Chengdu 611731, China; ygzhang@uestc.edu.cn

**Keywords:** magnetic, molecular imprinting polymers, cefixime, core–shell structure, adsorption

## Abstract

Novel core–shell magnetic molecularly imprinted polymers (MMIPs) were synthesized using the sol–gel method for the adsorption of cefixime (CFX). Fe_3_O_4_@SiO_2_ is the core, and molecularly imprinted polymers (MIPs) are the shell, which can selectively interact with CFX. The preparation conditions, adsorption kinetics, adsorption isotherms, selective adsorption ability, and reutilization performance of the MMIPs were investigated. The adsorption capacity of MMIPs for CFX was 111.38 mg/g, which was about 3.5 times that of MNIPs. The adsorption equilibrium time was 180 min. The dynamic adsorption experiments showed that the adsorption process of MMIPs to CFX conformed to the pseudo-second-order model. Through static adsorption study, the Scatchard analysis showed that MMIPs had two types of binding sites—the high-affinity binding sites and the low-affinity binding sites—while the Langmuir model fit the adsorption isotherms well (R^2^ = 0.9962). Cefepime and ceftiofur were selected as the structural analogs of CFX for selective adsorption studies; the adsorption of CFX by MMIPs was higher than that of other structural analogs; and the imprinting factors of CFX, cefepime, and ceftiofur were 3.5, 1.7, and 1.4, respectively. Furthermore, the MMIPs also showed excellent reusable performance.

## 1. Introduction

Cefixime (CFX), an important β-lactam ring antibiotic, is mainly used to treat throat infections, gonorrhea, and pneumonia [1,2]. Advantages such as strong antibacterial activity and good efficacy have enabled the wide use of CFX in the treatment of diseases in humans, poultry, and aquaculture [3,4]. However, its frequent use has raised concerns over harm to both the environment and human health. In the production process of CFX, the separation, extraction, purification, and other processes will produce high-concentration organic wastewater. CFX has been detected in the water and soil of many countries and regions; for example, high concentrations of CFX were found in hospital wastewater in Bangladesh [5], which is difficult to treat due to its strong polarity and biological enrichment [6]. At present, the removal technologies of CFX in the environment mainly include physical separation, biodegradation, chemical oxidation, etc. [7,8,9]. Vajihe Hasanzadeh et al. used metal hydroxide to activate jujube fruit residue at high temperature, which adsorbed CFX based on physical separation [10]. Physical separation is complex, requires high temperature, and has poor specificity for pollutants. Abotaleb Bay et al. developed a biofilm reactor for treating CFX in wastewater. The degradation rate could reach 70.9% at 92 mg/L, but the degradation efficiency was reduced to 34.8% at 122 mg/L [11]. In the process of biodegradation, there are disadvantages; for example, toxic substances may be produced, and rigorous temperature conditions are required. The operation cost of chemical oxidation is high, the operation is complicated, and toxic products may still be produced after degradation. For example, Hasani et al. degraded CFX in water via the ultrasonic and electric Fenton method, but the cost was high, the operation was complicated, and toxic products might still have been produced after degradation [12]. Therefore, it is necessary to design a low-cost material, which can identify CFX in complex systems.

MIPs are synthetic materials with a specific selection of target molecules [13], which, owing to their imprinted site, are complementary to the target molecules in shape and size [14,15], vividly described as artificially synthesized tailor-made polymers [16,17]. MIPs benefit from excellent properties, such as prominent selectivity, strong anti-interference ability, and convenient synthesis [18,19,20]. They have application opportunities in solid phase extraction, controlled drug release, sensors, and catalysis [21,22]. However, the use of traditional MIPs includes obstacles related to their low binding capacity and mass transfer [23,24,25]. By designing the molecular recognition sites on the surface of imprinted materials, the surface molecular imprinting technology improves the mass transfer between the recognition sites and target molecules, facilitates the elution and recombination of template molecules, improves the recognition efficiency and binding speed, and avoids the disadvantages of traditional methods.

The molecular imprinting technique of graft on the surface of a carrier has been extensively studied. The commonly used carriers include SiO_2_, TiO_2_, Al_2_O_3_, magnetic nanomaterials, etc. Shichao Ding et al. designed and synthesized peptide-imprinted mesoporous silica using a combination of the sol–gel method and molecular imprinting technology to specifically identify an immunostimulating hexapeptide from human casein; the adsorption capacity was 60.5 mg/g, and the imprinting factor was 4.51 [26]. In terms of the application effect, magnetic nanoparticles have received attention due to their good dispersion, controllability, small size, and excellent superparamagnetism [27,28]. MMIPs can not only specifically adsorb target molecules in a complex environment, but they can also be quickly separated by an external magnetic field, thus avoiding steps such as centrifugation, resulting in low cost, mild conditions, reducing material waste, and improving work efficiency [29,30]. Fatemeh Mirzapour et al. prepared the MMIPs of dextromethorphan via precipitation polymerization using Fe_3_O_4_@SiO_2_-C=C as the carrier and applied them to highly selective solid phase extraction; the recovery was 92–97%, and the adsorption capacity was 114.8 mg/g [31]. Chaoren Yan et al. used Fe_3_O_4_@SiO_2_ as the carrier and developed a combination of epigallocatechin-3-gallate (EGCG), imprinting technology, and magnetic nanoparticles to obtain a somewhat promising nanomaterial (MINs@EGCG) for amyloid inhibition, drug carrier, and facile separation triple functions; the cleansing efficiency was up to 80% [32]. Shikha Bhogal et al. prepared MMIPs for phthalate adsorption via surface imprinting using Fe_3_O_4_@SiO_2_ as a carrier; the recovery was 88.53–121.57%, with LOD ranging from 0.01 to 0.03 ng/mL [33]. Ziyang Lu et al. prepared a magnetic imprinted PEDOT/CdS nanoreactor for the adsorption and degradation of danofloxacin mesylate through the microwave-assisted surface imprinting technique, with a degradation rate of 84%; the adsorption capacity was 1.41 mg/g [34]. Mir Muhammad Gaho et al. prepared the MMIPs for norfloxacin adsorption through radical polymerization using oleic-coated Fe_3_O_4_ as the carrier, and the maximum adsorption capacity was 42.34 mg/g at 35 °C [35]. Ziyang Li et al. prepared the MMIPs for sulfamethoxazole using Fe-Mn impregnated peanut shell biochar as a functional monomer by using surface molecular imprinting technology, with a maximum adsorption capacity of 25.65 mg/g and an imprinting factor of 1.34 [36]. Notably, there are few reports on the adsorption of CFX on MMIPs, which is where our work was focused.

In this work, the sol–gel method was introduced to imprint molecules into the inorganic network structures to form a rigid structure. Compared with precipitation polymerization, radical polymerization, and other methods, this method is simple in operation, easy to control, and low cost [31,37,38,39]. Fe_3_O_4_@SiO_2_ was the core, and MIPs were used as the shell; silica was used as the intermediate carrier to connect the magnetic particles and the organic layer. CFX was used as the template, 3-aminopropyltriethoxysilane (APTES) as a functional monomer, and tetraethoxysilane (TEOS) as a crosslinker to be imprinted on the surface of Fe_3_O_4_@SiO_2_ with the sol–gel method. Thus, MMIPs with core–shell were prepared and showed high selectivity toward CFX.

## 2. Experimental Section

### 2.1. Materials

CFX, TEOS, cefepime, ceftiofur, and APTES were purchased from Aladdin Reagent Co., Ltd. (Shanghai, China). Crystalline sodium acetate, ethylene glycol, and polyethylene glycol 6000 (PEG) were purchased from Tianjin Zhiyuan Chemical Reagent (Tianjin, China). NH_3_·H_2_O (25%), methanol, ethanol, and acetic acid were purchased from Shanghai Chemical Reagent Co. (Shanghai, China).

### 2.2. Characterization

Infrared spectra were analyzed using Fourier transform infrared spectroscopy (FTIR; Tensor 27, Bruker, Billerica, MA, USA). The crystalline structures of the MMIPs were characterized via X-ray diffraction (XRD; D8, Bruker, Salbuluken, Germany). The magnetic properties were characterized through vibrating sample magnetometry (VSM; 7404, Lake Shore Company, Columbus, OH, USA). Morphological analysis was characterized through scanning electron microscopy (SEM; Sigma300, Zeiss, Berlin, Germany). Finally, the morphology of the MMIPs was characterized via transmission electron microscopy (TEM; JSM-7610FPlus, JEOL, Tokyo, Japan).

### 2.3. Preparation of MMIPs and Magnetic Non-Molecularly Imprinted Polymers (MNIPs)

#### 2.3.1. Preparation and Modification of Fe_3_O_4_

Fe_3_O_4_ was synthesized using the solvent-thermal method [40]. First, 2.025 g FeCl_3_·6H_2_O was dissolved in 60 mL ethylene glycol; then, 5.50 g NaAc and 1.50 g PEG were added, and the mixture was stirred magnetically. Finally, the mixture was sealed in the reactor at 190 °C for 8 h. The unreacted material was washed alternately with ultrapure water and ethanol and dried under a vacuum. The Fe_3_O_4_ nanoparticles were modified with SiO_2_ based on the hydrolysis of TEOS, according to the literature [41]. Subsequently, 0.2 g Fe_3_O_4_ was dispersed in a 125 mL solution of ethanol and ultrapure water (4:1, *v*/*v*), after which 1.8 mL NH_3_·H_2_O and 0.6 mL TEOS were added; the material was dried at 60 °C for 10 h in a vacuum to obtain Fe_3_O_4_@SiO_2_.

#### 2.3.2. Synthesis of MMIPs and MNIPs

Amounts of 0.135 g CFX, 0.420 mL APTES, and 30 mL methanol were mixed and stirred; then, amounts of 2.67 mL TEOS, 0.20 g Fe_3_O_4_@SiO_2_, and 1 mL acetic acid (1 mol/L) were added, and the mixture was stirred for 10 h. The mixture was then separated with magnets, washed repeatedly, and dried. A mixture of methanol/acetic acid (9:1, *v*/*v*) was used to elute CFX from the MMIPs until there was no UV-Vis adsorption at 288 nm. Then, the MMIPs were vacuum-dried at 60 °C for 10 h. The MNIPs were prepared without CFX but using the same protocol.

### 2.4. Binding Experiments

#### 2.4.1. Static Adsorption

An amount of 5 mg of MMIPs or MNIPs was added to a 10 mL CFX–methanol solution of 10–200 mg/L, which was shaken for 180 min; magnets were used for separation, and the absorbance of the supernatant was measured at 288 nm with UV-Vis. The adsorption capacity *Q* (mg/g) was calculated according to the following formula:

*Q* = (*C*_0_ − *C_e_*)*V*/*m* (1)

where *C*_0_ (mg/L) is the initial concentration of the CFX solution; *C_e_* (mg/L) is the equilibrium concentration of CFX; *V* (L) is the total volume; and *m* (g) is the weight of the MMIPs or MNIPs.

#### 2.4.2. Adsorption Kinetics

MMIPs or MNIPs (5 mg) were added to a 10 mL CFX–methanol solution of 200 mg/L and shaken at 25 °C for different times (30–240 min), and magnets were used for separation. The absorbance of CFX was measured using UV-Vis.

### 2.5. Selectivity

The selectivity of *MMIPs* was assessed using cefepime and ceftiofur as the structural analogs of CFX. *MMIPs* or *MNIPs* (5 mg) were added to 200 mg/L of methanol solution containing each compound. We used the imprinted factor (*IF*) to assess the selectivity of *MMIPs*, which is defined as

*IF* = *Q_MMIPs_*/*Q_MNIPs_* (2)

where *Q_MMIPs_* and *Q_MNIPs_* are the binding capacities of *MMIPs* and *MNIPs*, respectively.

### 2.6. Reusability

To evaluate the repetitive utilization rate, 5 mg of MMIPs was added to a 10 mL CFX–methanol solution of 200 mg/L and oscillated for 180 min, followed by magnetic field separation. The CFX in the MMIPs was washed with methanol/acetic acid (9:1, *v*/*v*), dried in a vacuum, and prepared for the next adsorption of CFX. The process was repeated five times.

## 3. Results and Discussion

### 3.1. Preparation of MMIPs and MNIPs

**Figure 1:**
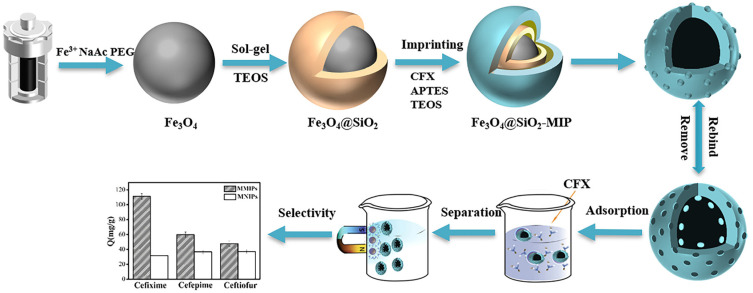
Schematic of the synthesis of CFX–MMIPs.

Fe_3_O_4_ nanoparticles exhibit a strong aggregation tendency and are easily oxidized in air [42,43]; therefore, we used Fe_3_O_4_ with silica modification. The synthesis process of MMIPs was as follows: (1) silica shell deposition on the surface of Fe_3_O_4_, (2) MIPs layer imprinting on Fe_3_O_4_@SiO_2_, and (3) removal of template molecules. The MIPs were imprinted on Fe_3_O_4_@SiO_2_ through the interaction of CFX, APTES, and TEOS. The carboxyl group of CFX interacts with the amino group of APTES to form a hydrogen bond; after eluting, the specific recognition sites for CFX are formed, ensuring that MMIPs can specifically recognize CFX. A schematic of the synthesis of CFX–MMIPs is shown in Figure 1.

### 3.2. Optimization of the Synthesis Conditions

#### 3.2.1. The Ratio of the Reactants

The ratio of the template molecule and crosslinker affects the property of MMIPs [44], including the stability of the recognized site and the mechanical strength of the polymer. The crosslinker binds the template molecule to the functional monomer, and an appropriate amount of crosslinker facilitates the formation of a rigid cavity for the adsorption of the target molecule. The amount of crosslinker is important; too little will render the synthesized polymer too soft to form stable affinity sites, while too much will cause an excessively high degree of crosslinking [45]. The excessive crosslinker will cover the recognition cavity and make it difficult for the elution of the template molecule, causing mass transfer resistance and adversely affecting the adsorption [46]. Therefore, to obtain better selective MMIPs, the ratio of the reactants should be carefully considered.

**Figure 2:**
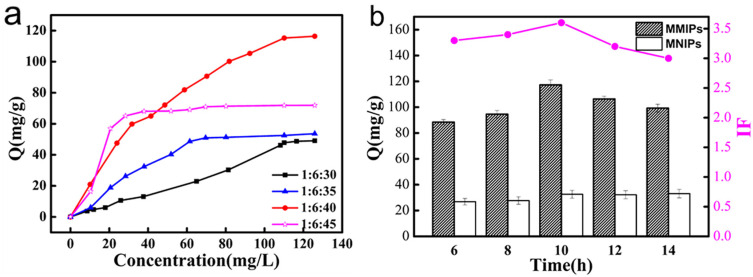
Effect of the crosslinker on the binding property of MMIPs: (**a**) The adsorption capacity of MMIPs and MNIPs at different polymerization times (**b**).

As shown in Figure 2a, the adsorption capacity first increased and then decreased with an increase in the TEOS ratio. When the ratio was 1:6:40, the adsorption capacity reached a maximum; when the ratio exceeded 1:6:40, the adsorption capacity was reduced, which could have resulted from excessive TEOS hindering the elution of the template molecule and blocking CFX from the recognition site. When the ratio was less than 1:6:40, the MMIPs exhibited poor performance in the adsorption capacity. Thus, for the rigid construction of MMIPs with a high binding capacity, the optimal molar ratio of CFX, APTES, and TEOS was 1:6:40.

#### 3.2.2. Influence of Polymerization Time

The polymerization time of the MMIPs significantly affected the adsorption capacity of CFX, and polymerization times of 6, 8, 10, 12, and 14 h were selected for investigation (Figure 2b). A polymerization time of less than 10 h resulted in a low adsorption capacity, possibly due to fewer imprinting sites. At a polymerization time of 10 h, the adsorption capacity reached its maximum. However, a polymerization time longer than 10 h resulted in a decreased adsorption capacity, possibly due to CFX having difficulty reaching the imprinted sites. Therefore, the optimum polymerization time was 10 h.

### 3.3. Characteristics of MMIPs and MNIPs 

**Figure 3:**
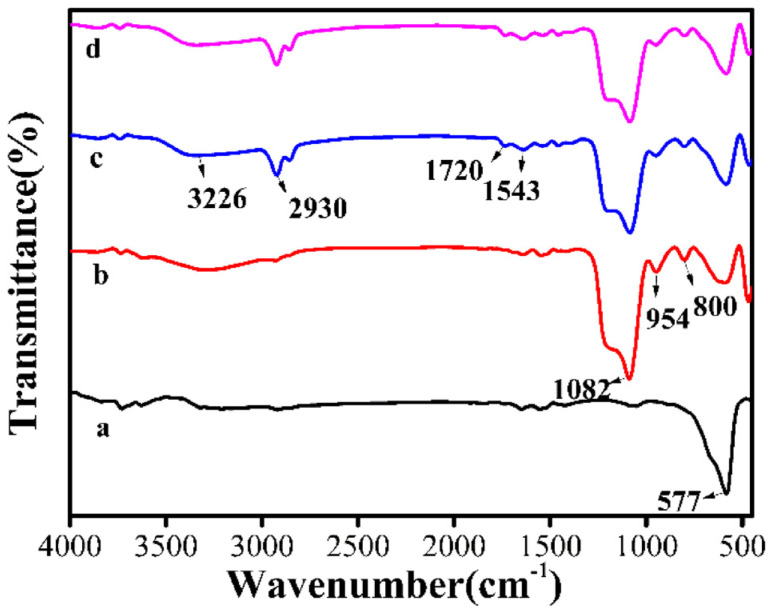
FTIR spectra of Fe_3_O_4_ (a), Fe_3_O_4_@SiO_2_ (b), MMIPs (c), and MNIPs (d).

The FTIR spectra of Fe_3_O_4_, Fe_3_O_4_@SiO_2_, MMIPs, and MNIPs (Figure 3) show a peak at 577 cm^−1^, which is the typical band of Fe_3_O_4_ [47] (Figure 3a). The new peak at 1082 cm^−1^ in the spectrum of Fe_3_O_4_@SiO_2_ is attributed to Si–O–Si, and the peaks at 954 cm^−1^ and 800 cm^−1^ represent the vibration absorption of the Si–O bond in Si–OH and the bending vibration absorption peak of Si–O–Si, indicating the successful synthesis of Fe_3_O_4_@SiO_2_ (Figure 3b). Meanwhile, the characteristic peaks of the N–H bond at 1543 cm^−1^, Si–O–Si and Si–O–H bonds at 1082 cm^−1^, and C–H stretching at 2930 cm^−1^ are the results of the imprinted layer being successfully bonded to the surface of Fe_3_O_4_@SiO_2_ by reacting with CFX, APTES, and TEOS [48]. Peaks at 1720 cm^−1^ (C=O) and 3226 cm^−1^ (O-H) were observed, indicating the presence of carboxyl groups in the MMIPs [49]. The characteristic absorption peaks of the MMIPs and MNIPs were not significantly different (Figure 3c,d), indicating that the addition of template molecules did not change the main functional groups of the polymer.

**Figure 4:**
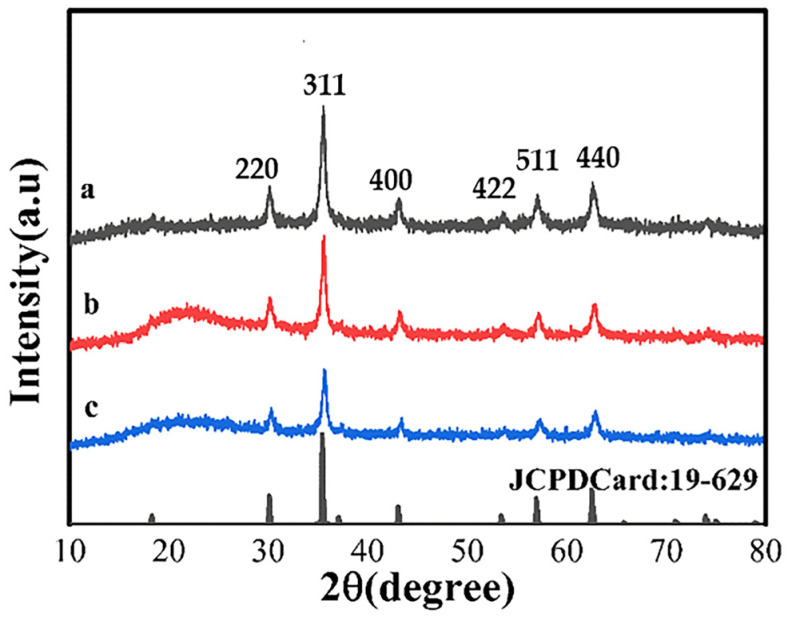
XRD pattern of Fe_3_O_4_ (a), Fe_3_O_4_@SiO_2_ (b), and MMIPs (c).

Figure 4 depicts the XRD pattern of Fe_3_O_4_, Fe_3_O_4_@SiO_2_, and MMIPs. The characteristic XRD absorption peaks of Fe_3_O_4_ appeared at 2θ = 30.38°, 35.58°, 43.14°, 53.48°, 57.08°, and 62.66°, corresponding to the stereoscopic crystal planes (220), (311), (400), (422), (511), and (440) of Fe_3_O_4_, respectively. This is consistent with the JCPDS-International Centre (JCPDSCard: 19-629) and proves that the prepared product was Fe_3_O_4_. When 2θ = 22°, there is a wider diffraction peak corresponding to the amorphous SiO_2_ in Fe_3_O_4_@SiO_2_ (Figure 4b). The weakening of the peak at 2θ = 22° was due to an imprinting layer on the Fe_3_O_4_@SiO_2_ surface (Figure 4c). The distinguishable characteristic diffraction peaks of Fe_3_O_4_ were observed for the three samples, indicating that the crystal structure of Fe_3_O_4_ remained unchanged during the imprinting process and was incorporated into all the samples.

**Figure 5:**
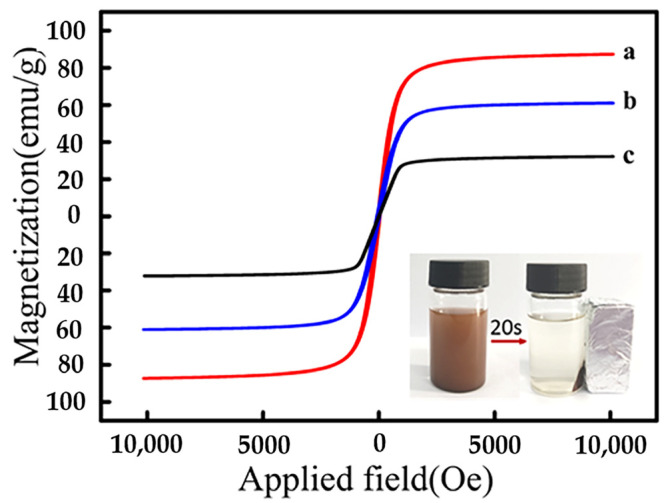
Magnetic hysteresis loop of Fe_3_O_4_ (a), Fe_3_O_4_@SiO_2_ (b), MMIPs (c), and the inserted figure depicts MMIPs dispersed in solution (left) and collected by an external magnet (right).

The magnetic properties of synthetic materials were analyzed using VSM. As illustrated (Figure 5), the synthetic materials crossed the zero point, showing superparamagnetic properties with almost no coercivity or remanence, indicating that the particles could be dispersed in a short time and exhibited a strong response to the magnetic field. The saturation magnetization (Ms) values of Fe_3_O_4_, Fe_3_O_4_@SiO_2_, and MMIPs were 87.2 emu/g, 61.0 emu/g, and 32.2 emu/g, respectively. The Ms of MMIPs showed a slight decrease compared to Fe_3_O_4_, which was attributed to the shielding effect of the Fe_3_O_4_ surface parcel Si coating and the molecularly imprinted layer. However, the MMIPs were still magnetic enough to meet the requirements of an effective magnetic carrier and could be separated by external magnets. As shown in the inset of Figure 5, the MMIPs were attracted to the bottle wall within 20 s, and the dispersed liquid became transparent, further verifying the successful synthesis and excellent magnetic properties of MMIPs.

**Figure 6:**
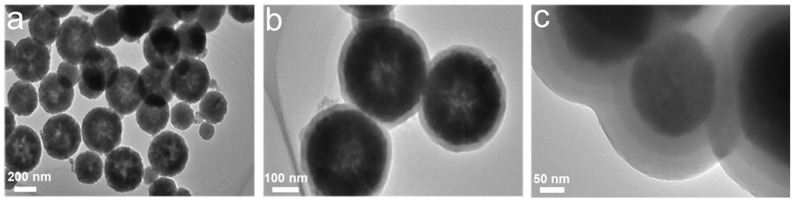
TEM of Fe_3_O_4_ (**a**), Fe_3_O_4_@SiO_2_ (**b**), and MMIPs (**c**).

The synthetic materials were characterized by TEM, and the image of Fe_3_O_4_ (Figure 6a) shows a uniform size distribution with good dispersion and no obvious agglomeration. After silanization, the particle size changed significantly, corresponding to the approximately 50 nm layer of silica evenly coating the surface of Fe_3_O_4_ (Figure 6b), providing evidence that Fe_3_O_4_ was completely and uniformly coated by silica. After imprinting CFX as the template, the imprinting layer was observed to be approximately 60 nm (Figure 6c), which may have been caused by the combined reaction of organic compounds on the particle surface. These observations initially confirmed that the MMIPs were prepared.

**Figure 7:**
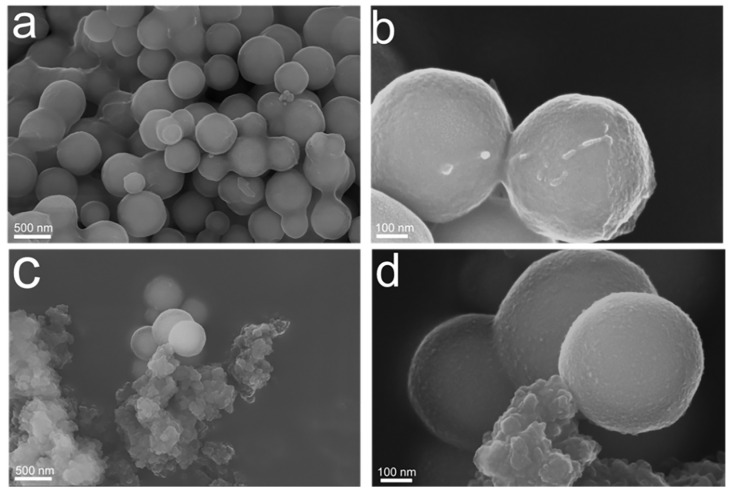
SEM of MMIPs (**a**,**b**) and MNIPs (**c**,**d**).

We used SEM to intuitively observe the surface morphology characteristics, and Figure 7 shows the morphological structures of MMIPs and MNIPs. The imprinted polymer surface became rough and uneven, indicating tiny “cavities” in the polymer surface and illustrating the deposition of the polymers over the surface of Fe_3_O_4_@SiO_2_. The rough surface of the polymer, which improves the adsorption capacity and recognition ability of the template, resulted from the remaining imprinted cavities after template elution. The MMIPs and MNIPs exhibited surface differences, where the latter had a relatively smooth and flat surface. This further elucidated the differences in adsorption effects, as the MMIPs had cavities, which could specifically recognize CFX, and thus, better adsorption.

### 3.4. Adsorption Properties of MMIPs and MNIPs

#### 3.4.1. Dynamic Adsorption

**Figure 8:**
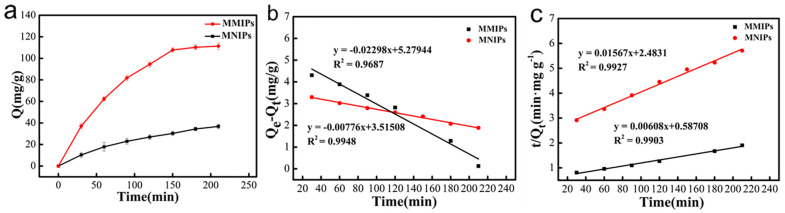
Kinetic adsorption curve of MMIPs and MNIIPs (**a**): Fitting using a pseudo−first−order (**b**) and pseudo−second−order (**c**) kinetic model for the binding CFX.

The adsorption kinetics of MMIPs and MNIPs were determined (Figure 8a), revealing that MMIPs could rapidly adsorb CFX in less than 150 min, after which the rate of adsorption capacity growth gradually decreased, finally reaching saturation after 180 min. Since there were imprinted sites on the surface of MMIPs at the initial stage of adsorption, CFX was rapidly adsorbed. After 180 min, an increasing number of binding sites were occupied, and the mass transfer of CFX in solution to the internal pores was subject to resistance. The adsorption capacity of MMIPs was approximately 3.52 times higher than MNIPs because CFX was not involved in the preparation of MNIPs; as such, there were no specific recognition holes or imprinting sites. The adsorption of CFX by MNIPs was mainly caused by non-specific adsorption of van der Waals force and other forces.

To study the mass transfer mechanism of polymer-adsorbed CFX, the equilibrium data were fitted using the two models of pseudo-first-order and pseudo-second-order binding as follows [50]:

ln(Q_e_ − Q_t_) = lnQ_e_ − k_1_t (3)

(4) $$\frac{t}{Q_{t}}=\frac{t}{Q_{e}}+\frac{1}{k_{2}Q_{e}^{2}}$$

where Q_e_ is the adsorption equilibrium capacity of MMIPs or MNIPs, and Q_t_ is the adsorption capacity at time t; t (min) is the adsorption time; and k_1_ (min^−1^) and k_2_ (mg g^−1^ min^−1^) are the pseudo-first-order and pseudo-second-order rate constants of adsorption, respectively.

The fitted data are shown in Table 1; the pseudo-second-order model (Figure 8c) was better than the pseudo-first-order model (Figure 8b). The adsorption process by MMIPs conformed to the pseudo-second-order model, and the adsorption process was controlled by chemisorption.

#### 3.4.2. Static Adsorption

**Figure 9:**
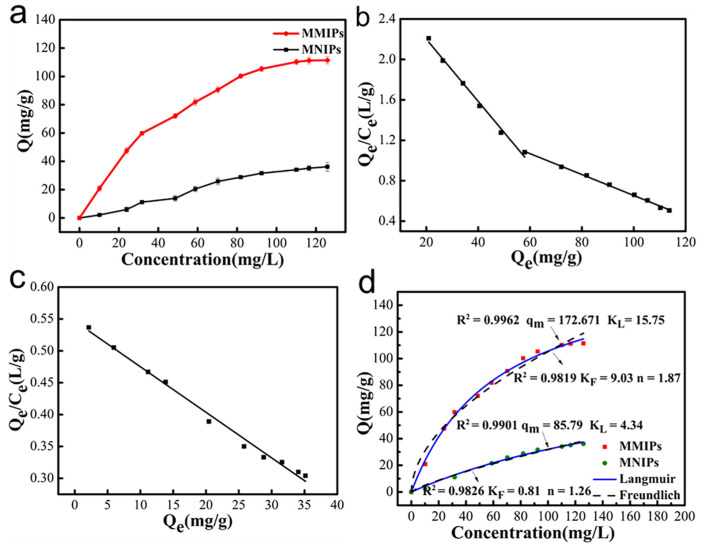
Adsorption isotherm of MMIPs and MNIPs (**a**); Scatchard plot analysis of CFX binding onto MMIPs (**b**) and MNIPs (**c**); Langmuir and Freundlich adsorption isotherms of CFX onto MMIPs and MNIPs (**d**).

The adsorption isotherms (at 25 °C) are shown in Figure 9a. At the same concentration, the adsorption capacity of MMIPs was higher than that of MNIPs, and the maximum adsorption capacity of MMIPs was 111.38 mg/g, which indicated that MMIPs had specific adsorption sites for CFX compared with MNIPs.

To study the specific binding properties between MMIPs and CFX, Scatchard analysis was used to analyze the binding data. The results of the Scatchard analysis are shown in Table 2, and the Scatchard equation is as follows:

(5) $$\frac{Q_{e}}{C_{e}}=\frac{Q_{m}}{K_{d}}-\frac{Q_{e}}{K_{d}}$$

where Q_e_ (mg/g) is the adsorption capacity of the MMIPs at equilibrium; Q_m_ (mg/g) is the maximum adsorption capacity; C_e_ (mg/L) is the concentration of the supernatant at equilibrium; and K_d_ (mg/L) is the equilibrium dissociation constant.

As shown in Figure 9b, there were two distinct linear sections, indicating that two types of binding sites with different binding properties existed on the surface of the MMIPs. This is because a variety of complexes with different stabilities can be formed between CFX and APTES, and different types of complexes form binding sites with different adsorption properties during polymerization. K_d1_ and Q_max1_ for the linear section on the left were calculated to be 32.542 mg/L and 91.6043 mg/g, respectively, and those for the linear section on the right were calculated to be 96.618 mg/L and 163.217 mg/g, respectively. Because K_d1_ < K_d2_, the equation on the left corresponds to the high-affinity binding sites of MMIPs, while the equation on the right corresponds to the low-affinity binding sites of MMIPs. In contrast, the Scatchard fitted curve of the MNIPs (Figure 9c) shows only one straight line, which indicates that there was only one non-specific recognition site in the MNIPs.

The binding data were analyzed using the Langmuir and Freundlich isotherm models to assess the maximum adsorption capacities of MMIPs and MNIPs. The Langmuir isotherm model describes a monolayer adsorption process and assumes that adsorption occurs at specific and uniform adsorption points within the adsorbent [51].

The Freundlich model is suitable for multilayer adsorption [52]. The Freundlich and Langmuir equations are, respectively, expressed as follows [22]:

(6) $$Q_{e}=K_{F}C_{e}^{1/n}$$

(7) $$Q_{e}=\frac{Q_{m}K_{L}C_{e}}{1+K_{L}C_{e}}$$

where Q_e_ and Q_m_ are the equilibrium and maximum adsorption amounts of MMIPs (mg/g), respectively; C_e_ is the equilibrium concentration of CFX (mg/L); K_L_ is the Langmuir model constant; and K_F_ and n are the Freundlich model constants (mg/L).

The correlation constants were calculated using the Freundlich and Langmuir models. The Langmuir isotherm was well fitted and could better describe the binding process (Figure 8d). It could therefore be inferred that the adsorption of CFX on MMIPs was monolayer adsorption.

### 3.5. Specificity

**Figure 10:**
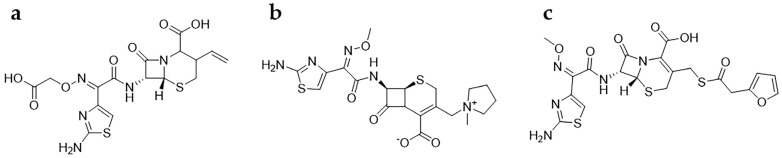
Structures of cefixime (**a**), cefepime (**b**), and ceftiofur (**c**).

**Figure 11:**
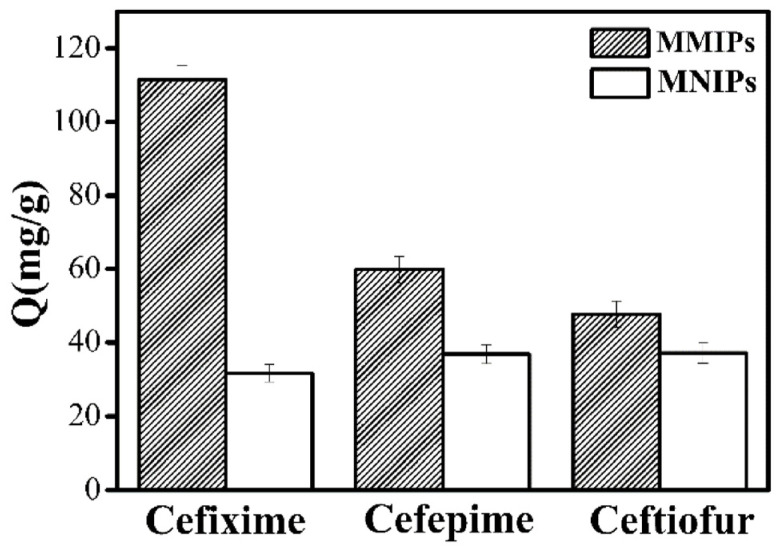
Adsorption capacities of CFX and its structural analogs.

The adsorption selectivity of MMIPs for CFX was studied. Cefepime and ceftiofur were selected as the structural analogs of CFX (Figure 10). As shown in Figure 11, the adsorption of CFX by MMIPs was higher than that of the other structural analogs, indicating a high selectivity of MMIPs for CFX. For MNIPs, however, the adsorption of CFX and the other analogs was not significantly different. The IF values of CFX, cefepime, and ceftiofur were 3.5, 1.7, and 1.4, respectively.

### 3.6. Reusability

**Figure 12:**
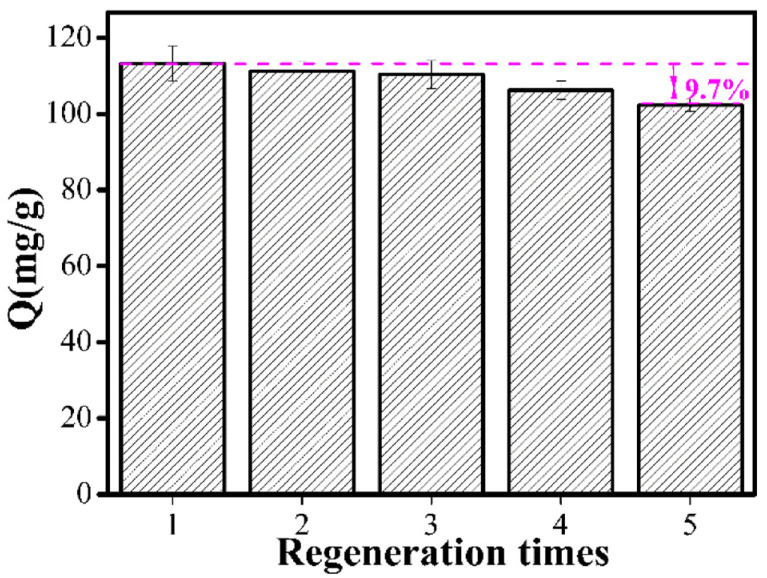
Regeneration properties of MMIPs.

The reusability of MMIPs is important for economical, reliable, and sustainable applications. Thus, five adsorption–desorption cycles were investigated (Figure 12). The adsorption capacity of MMIPs decreased by 9.7% with repeated use. The decrease may have been due to the destruction of imprinting recognition sites through multiple adsorption–desorption cycles, showing that the adsorption capacity remained high after multiple applications and indicating that the MMIPs had outstanding regeneration. Therefore, the MMIPs exhibited strong potential for practical applications.

## 4. Conclusions

Using CFX as a template molecule, APTES as a functional monomer, and TEOS as a crosslinker, a surface-molecule-imprinted polymer with specific adsorption of CFX was synthesized on the surface of Fe_3_O_4_@SiO_2_ via surface imprinting technology, which could achieve rapid separation under the external magnetic field. The adsorption performance of MMIPs was evaluated through adsorption kinetics, adsorption isotherm, and reusability. The results showed that MMIPs could reach the adsorption equilibrium within 180 min, with a good imprinting effect and selectivity (imprinting factor 3.5), high adsorption capacity (111.38 mg/g), and excellent reuse performance (after five cycles of utilization, the adsorption capacity of cefixime could still be maintained at 90.3%). The synthesized molecularly imprinted polymer can be used as a new adsorption material, with high selectivity and high adsorption capacity for cefixime.

## Figures and Tables

**Table 1:** Adsorption kinetic constants of pseudo-first-order and pseudo-second-order models for MMIPs and MNIPs.

Materials	Q_e_,_exp_ (mg g^−1^)	Pseudo-First-Order Model			Pseudo-Second-Order Model		
		Q_e_,_cal_ (mg g^−1^)	K_1_ (min^−1^)	R^2^	Q_e_,_cal_ (mg g^−1^)	K_2_ (mg g^−1^ min^−1^)	R^2^
MMIPs	111.38	196.25	0.0230	0.9687	164.47	0.0063	0.9903
MNIPs	36.15	27.52	0.0070	0.9948	64.10	0.0010	0.9927

**Table 2:** Results of the Scatchard analysis.

Materials	Binding Site	Linear Equation	k_d_ (mg/L)	Q_max_ (mg/g)
MMIPs	Higher affinity site	Q/C_e_= −0.0307 Q + 2.815 (R^2^ = 0.9914)	32.542	91.604
		Q/C_e_= −0.0103 Q + 1.689 (R^2^ = 0.9972)	96.618	163.217
MNIPs	Lower affinity site	Q/C_e_= −0.0071 Q + 0.546 (R^2^ = 0.9960)	39.860	76.443

## Data Availability

The data presented in this study are available on request from the corresponding author.
